# Retrospective Analysis of Echinococcosis in an Endemic Region of Turkey, a Review of 193 Cases

**Published:** 2010-09

**Authors:** S Gulsun, B Cakabay, M Nail Kandemır, S Aslan, B Atalay, N Sogutcu, O Satıcı, M Kangın

**Affiliations:** 1 Department of Infectious Diseases and Clinical Microbiology, Diyarbakir Education and Research Hospital, Diyarbakir, Turkey; 2 Department of General Surgery, Diyarbakir Education and Research Hospital, Diyarbakir, Turkey; 3 Department of Thoracic and Vascular Surgery, Diyarbakir Education and Research Hospital, Diyarbakir, Turkey; 4 Department of Radiology, Diyarbakir Education and Research Hospital, Diyarbakir, Turkey; 5 Department of Pathology, Diyarbakir Education and Research Hospital, Diyarbakir, Turkey; 6 Department of Statistics, Dicle University, Diyarbakir, Turkey; 7 Department of Pediatrics, Diyarbakir Education and Research Hospital, Diyarbakir, Turkey

**Keywords:** Echinococcosis, Lung, Liver, Turkey

## Abstract

**Background:**

The hydatidosis is endemic in our region. Some of the recent studies revealed that hydatid cyst prevalence is decreasing gradually in Turkey. The aim of this study was to investigate the actual prevalence of hydatidosis in an endemic region of Turkey, and to share our experiences in the medical and surgical management of hydatidosis.

**Methods:**

Data were collected retrospectively from the records of 193 patients who had a diagnosis of hydatidosis, and admitted to Diyarbakir Education and Research Hospital. Imaging techniques, histology and serology were used for diagnosis.

**Results:**

From records of 772 cystic patients whose cysts were localized in the lung and liver, 193 (25%) of them were diagnosed with cyst hydatidosis. Lung hydatidosis was found statistically significant among these cases (Chi-square=24.88, *P*< 0.0001). Postoperative recurrence was detected in seven (3.62%) patients. All postoperative recurrences were observed in the consequent three years period.

**Conclusion:**

The prevalence of hydatidosis is still high in southeast Turkey and not only in children but also in adult cases in our region lung hydatidosis is frequent corresponding with other organ hydatidosis. We also found that the most risky period in recurrence rates is the consequent post-operative three years. According to our experiences, transthoracic approach in lung hydatidosis, external drainage, and cystectomy in liver hydatidosis is safe and effective choices in surgical treatment.

## Introduction

Cystic Echinococcosis (CE) has for many years been a manifestation of parasitic infection, which can potentially lead even to death (1). Human echinococcosis remains a significant health problem for the Mediterranean countries (2). Hydatid disease may be located in any organ of the body. The organ that is involved most frequently is the liver (50% to 70%), with the lung being the second most common site (20% to 30%) (3, 4). However, the brain, heart, bone, and muscle may be involved as well. Clinical signs and symptoms of CE depend on the localization, size, and relationships with the adjacent organs and complications (3–5).

In this study, we present a retrospective and epidemiologic analysis of echinococcosis in an endemic region covering 4 years period.

## Material and Methods

### Study settings and patients

**Fig. 1:**
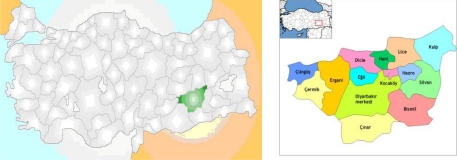
A (Left) Location of Diyarbakir in Turkey and B (Right) Diyarbakir districts

Diyarbakir is one of the largest city in southeastern Turkey (Fig. 1) situated on the banks of the River Tigris, is the administrative capital of the Diyarbakir Province with a population of almost 1.5 million people. Diyarbakir Education and Research Hospital is the only and the biggest state hospital serves the region. We retrospectively investigated the records and films of 193 patients with hydatid disease, who were treated in the Department of Thoracic and Vascular Surgery, General Surgery and Infectious Diseases at Diyarbakir Education and Research Hospital in Turkey between July 2005 and June 2009. Information on history, clinical manifestations, radiological findings, methods, and results of surgery were analyzed. This study was approved by the Local Health Directorate and Administrative Committee of Diyarbakir Education and Research Hospital. Serologic tests (Echinococcal indirect hemagglutination test), Ultrasonography (USG), chest and anterioposterior graphies, pathologic examination, Computed tomography (CT) and nuclear magnetic resonance spectroscopy (MR) were used for the diagnosis. Patients who could be contacted during our review were asked to return to the hospital for indirect hemagglutination test (IHA), anterioposterior graphies, USG and CT scan to evaluate disease recurrence during the follow-up period.

### Statistics

Statistical analysis was carried out by using the SPSS 15.0 program (SPSS 15.0 for Windows Evaluation Version Release 15.0; 06 September 2006). Chi-square test was used in the evaluation of different variables. *P*< 0.05 was considered statistically significant.

## Results

The group consisted of 98 (50.77%) male and 95 (49.23%) female patients. We found no significant correlation between organ involvements and the gender of patients (Chi-square=1.122, *P*=0.29).

**Fig. 2:**
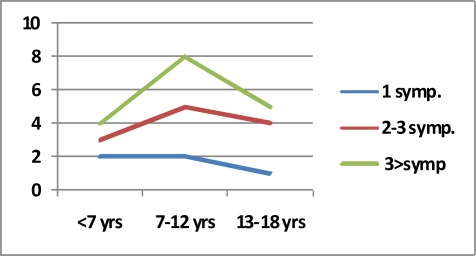
Number of symptoms by age in children groups

The age intervals were between 3–88 years (Mean: 23 yr). Twenty-eight patients were younger than 16 (14.5%). We investigated the cyst hydatid cases in two groups. First was the children group and the ages were divided into three; pre-education (0–6 years), primary school (7–12 years) and high school period (13–18 years). In the second group, the ages were divided as; young (18–25 years old), adult (26–45) and middle age (>45 years old). There was no significant correlation between the age groups in children and the number of symptoms (Chi- square=0.18) (Fig. 1), and no significant correlation was found between the age groups in adults and the number of symptoms (Chi-square=4.434) (Fig. 2).

Seven hundred seventy-two patients had lung and liver cysts, 193 out of 772 patients (25%) were diagnosed with hydatid cyst disease caused by *Echinococcus granulosus* in the location of liver and/ or lung during July 2005– June 2009 in Diyarbakir Education and Research Hospital. One hundred ninety-one patients underwent surgery; all patients (193) received medical treatment.

Cysts were found in the lungs and livers of 138 (71.5%) and 47 cases (24.4%), respectively with 3 (1.5%) cases having simultaneous liver and lung cysts and 5 (2.5%) cases with simultaneous liver/ lung and other organ (myocardial, over, bone) cysts. Seventy-four patients had right lung, 2 had bilateral, the other 62 had cysts on the left lung, whereas 47 (24.3%) had cysts in the liver. Lung involvement was found statistically significant in hydatid cyst cases (Chi-square=24.88, *P*<0.0001).

**Fig. 3:**
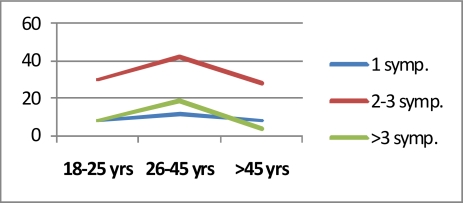
Number of symptoms by age in adults groups

Clinical manifestations varied widely depending on the status of the hydatid cysts. The significant clinical manifestations of pulmonary hydatid cysts were chest pain and cough (47% and 72% respectively). Right-upper abdominal pain and fever were the most common symptoms in cases with hepatic cysts (68% and 62% respectively)**.** The significant clinical manifestations of pulmonary hydatid cyst in children (smaller than 16 years of age) were cough (75%), dyspnea (64%) and fever (50%)**.** Cough was found to be a statistically significant symptom in adult group (Chi-square=14.85, *P*<0.001) and in the group smaller than 16 years of age (Chi-square=9.81, *P*<0.05). There was no important correlation between the number of symptoms according to the site (Chi-square=0.72) and the localization of the hydatidosis (Chi-square 1.52) (Table 1).

**Table 1:** The relationship between the number of symptoms, the localization of the cysts and the site of organ involvements

No. of Symptoms	The site of organ involvements			The localization of the cysts		
	Right	Left	Total	Lung	Liver	Total
**1**	28	25	53 (28.3%)	44	11	55 (29.7%)
**2–3**	41	32	73 (39%)	52	18	70 (37.8%)
**>3**	37	24	61 (32.6%)	42	18	60 (32.4%)
**Total**	106 (56.7%)	81 (43.3%)	187 (100%)	138 (74.6%)	47 (25.4%)	185 (100%)

Ruptured hydatid cysts were observed in 18 patients on admission (9.3%). Postoperative recurrence of hydatid disease was detected in 7 cases (3.6%). All postoperative recurrences were observed in the subsequent three years period. Five patients had a second incidence of hydatidosis and 2 of them a third one.

The most common laboratory findings were anemia (58%), high sedimentation rate (54%) and elevated liver enzymes (34%). Leucocytosis referred to an infected perforated cyst (62%). There was an important correlation between ruptured cyst and presence of three or more clinical symptoms (100%).

Patients who participated in our study (49.7%) were from rural parts of our region and 100 (51.8%) were Green Card holders who had low income/ year. In the diagnosis and during the follow-up period, chest roentgenogram was performed for all patients (100%). In liver hydatidosis, USG was used in 43±3 (92%) cases, while roentgenograms were used only in 18±3 (38%) cases.

Surgical approach involved thoracotomy, cystotomy, enucleation, wedge resection, capitonnage and segmentectomy resection techniques in lung hydatidosis. In perforated hydatidosis intrapleural lavage, drainage of the pleural cavity and atelectatic segment resection added to the applied surgical approach. In liver hydatidosis; cyst evacuation, complete pericystectomy, omentopexy, segmentectomy, drainage, capitonnage and lobectomy resection techniques were used.

The postoperative course was regular with no mortality and no major morbidity. Postoperative complication was detected only in 4 patients (2%). Postoperative complications were postoperative atelectasy (n=2), biliary fistula (n=1), and bronchopleural fistula (n=1). Antihelminthic benzimidazoles (albendazole, 800 mg/d in adults, 10 to 15 mg/kg/day in children, in two divided doses for the first 15 days of the month, before 1 month of surgery and 4 months after surgery, for a treatment period of 5 months) applied to the patients admitted to the Department of Infectious Diseases.

## Discussion

Hydatidosis is a zoonosis of worldwide distribution mainly caused by the metacestode *E. granulosus* (6). The aim of this study was to establish the actual prevalence of cyst hydatid in Diyarbakir and in its districts, also to share four different branch clinic doctors’ observations on hydatidosis and the success of medical and surgical treatment of hydatid cyst.

The hydatidosis is endemic in our region. We found the prevalence of echinococcosis as 25% in correspondence of all cystic diseases in our region. Seimenis (2) overviewed the epidemiological situations on *Echinococcus* in the Mediterranean region and determined the factors favoring transmission of cystic echinococcosis. These conditions are similar with our region's conditions such as, most of rural families live in close proximity to their sheep, goats, cattle and dogs, large number of free-roaming dogs, home slaughter, uncontrolled population movement and in addition to these factors the traditional meal consumed frequently in our region, cig kofte, prepared with uncooked meat.

Ninety-six patients (49.7%) were living in the rural areas of Diyarbakir (Ergani, Hani and villages) and its peripheral zones. 51.8% patients of hydatidosis were green card holders and had low income/year (cut-off for low income degree is 250 USD/ month earning). The Green Card is a special card covering medical drug expenses but excluding the cost of out patient drugs. This card is directly funded by the Turkish Government and financing health care for the poor in Turkey.

In our study, there were 98 males (50.7%) and 95 females (49.2%), aged 3–88 (mean: 23) years. There was an equal sex distribution in cases; we assume that the cause of equality is due to the lifestyles and habits of people living in this region. Women generally involve in farming and animal care more than men in region do. The mean age was also very young in our study, it was due to the crowded families living together and especially in rural parts, each family has at least 4 children.

In our study, the most affected organ was lung (71.5%). This is in contrast with most of the studies that the liver found the most affected organ (6–8). Generally, the liver in adults and the lungs in children are the predominant sites (6). The lung predominance may be due to geographical differences in the distribution of the organ involved related to some biological factors in the parasite or host also cysts grow faster in compressible organs such as the lung, and this may account for the relatively high incidence of the disease in this organ (9). The mean age is very young in our study and this may be the other cause of lung predominance.

9.3% of lung hydatid cases were admitted to the hospital with ruptured hydatid cysts and 3.6% of them presented with recurrences. In spite of the ruptured cases (9.3%), the recurrence rate (3.6%) is low in our series. Five patients in our series had a second incidence of hydatidosis and two of them a third one. In other studies, the rate of recurrences after lung and/or liver hydatid surgery has been reported to range from 0– 12% (9, 10). In our study, all recurrence cases following surgery observed in the consequent three years. According to Akyildiz et al. (11), recurrence may become symptomatic 3 to 4 years after surgery and they suggested that postoperative early USG profile and annual USG examination must be performed for at least 5 years to prevent misinterpretation in doubtful cases.

Intrathoracic and extra-pulmonary hydatid cysts may cause general symptoms and/ or compression symptoms in adjacent organs. Most of these symptoms are similar to other cystic lesions. The significant clinical manifestations of lung hydatid cysts in all ages (children, adult) were chest pain, cough, and dyspnea. In liver hydatidosis, abdominal pain (68%) and fever (62%) were the most common symptoms. Isitmangil et al. (12) found the most common clinical symptom as cough (51%). Dakak et al. (13) reported that the common clinical symptoms were cough (56%), chest pain (52%), and fever (34%). In most of the studies, authors mentioned that at least 2 symptoms can mainly be observed besides the general symptoms of cyst hydatid (9, 12, 13).

Briefly, an epidemiologic history and a cystic image may be sufficient in diagnosis of cyst hydatid disease (14). According to our study, chest X- rays in lung, USG in liver hydatidosis are the most helpful diagnostic tools for the diagnosis of recurrent hydatid disease. Serology used in the diagnosis of only 12% cases. This conclusion shows that, serology is not so helpful in diagnosis. Computed tomography and/ or MRI techniques are useful in the absence of a pathognomonic clinical and radiologic picture but these diagnostic tools are expensive for routine use. Hydatid disease may recur as the patient returns to the endemic area so that in the diagnosis of recurrent liver hydatid disease, examination with roentgenogram and USG with an epidemiologic history will be enough to evaluate the case as cystic hydatid disease. Pathologic examination is also very helpful in surgical cases.

Dogan et al. (15) reviewed 1,055 patients treated surgically for pulmonary hydatid disease and according to their report, postoperative complications occurred in 37 patients (3.5%) and the mortality rate was 1.7%. In our study, postoperative complication was 4 (2%) and mortality rate was 0%. As a conclusion, conservative surgery plus medical therapy is a good choice of treatment in hydatidosis.

In contrast with the other studies, the prevalence of hydatidosis has not decreased in southeast part of Turkey and hydatidosis is still an important public health problem in the pediatric and adolescent age groups especially in rural areas of Turkey (especially eastern and central regions of Anatolia) (16). In our study, we found lung hydatidosis prevalence more than liver hydatidosis in our region not only in children but also in adult cases. As postoperative complications and mortality rate is low, we recommend surgery combining anti-scolicidal therapy in the treatment of hydatidosis. A close follow-up and awareness of the patients is also very important in the prevention of recurrences. Transthoracic approach in lung hydatidosis, external drainage, and cystectomy in liver hydatidosis is safe and good choices in surgical treatment.

In conclusion, appropriate randomized controlled studies are needed to establish the definite medical and surgical management of hydatidosis.
